# Sjögren-Like Lacrimal Keratoconjunctivitis in Germ-Free Mice

**DOI:** 10.3390/ijms19020565

**Published:** 2018-02-13

**Authors:** Changjun Wang, Mahira Zaheer, Fang Bian, Darin Quach, Alton G. Swennes, Robert A. Britton, Stephen C. Pflugfelder, Cintia S. de Paiva

**Affiliations:** 1 Eye Institute of Zhejiang University School of Medicine, Zhejiang Provincial Key Lab of Ophthalmology, Hangzhou 310009, China; wangchangjun@zju.edu.cn; 2 Ocular Surface Center, Department of Ophthalmology, Cullen Eye Institute, Baylor College of Medicine, Houston, TX 77030, USA; mzaheer@bcm.edu (M.Z.); bftongji@hotmail.com (F.B.); stevenp@bcm.edu (S.C.P.); 3 Center for Metagenomics and Microbiome Research, Department of Molecular Virology and Microbiology, Baylor College of Medicine, Houston, TX 77030, USA; darin.quach@gmail.com (D.Q.); robert.britton@bcm.edu (R.A.B.); 4 Center for Comparative Medicine and Department of Molecular Virology and Microbiology, Baylor College of Medicine, Houston, TX 77030, USA; alton.swennes@bcm.edu

**Keywords:** fecal transplant, germ-free mice, dry eye, Sjögren syndrome, goblet cell, commensal bacteria

## Abstract

Commensal bacteria play an important role in the formation of the immune system but their role in the maintenance of immune homeostasis at the ocular surface and lacrimal gland remains poorly understood. This study investigated the eye and lacrimal gland phenotype in germ-free and conventional C57BL/6J mice. Our results showed that germ-free mice had significantly greater corneal barrier disruption, greater goblet cell loss, and greater total inflammatory cell and CD4^+^ T cell infiltration within the lacrimal gland compared to the conventionally housed group. A greater frequency of CD4^+^IFN-γ^+^ cells was observed in germ-free lacrimal glands. Females exhibited a more severe phenotype compared to males. Adoptive transfer of CD4^+^ T cells isolated from female germ-free mice into RAG1KO mice transferred Sjögren-like lacrimal keratoconjunctivitis. Fecal microbiota transplant from conventional mice reverted dry eye phenotype in germ-free mice and decreased CD4^+^IFN-γ^+^ cells to levels similar to conventional C57BL/6J mice. These findings indicate that germ-free mice have a spontaneous lacrimal keratoconjunctivitis similar to that observed in Sjögren syndrome patients and demonstrate that commensal bacteria function in maintaining immune homeostasis on the ocular surface. Thus, manipulation of intestinal commensal bacteria has the potential to become a novel therapeutic approach to treat Sjögren Syndrome.

## 1. Introduction

There is increasing evidence indicating that the microbiome—the bacterial communities that inhabit the human body—plays key roles in the development and function of the immune system. Virtually all surfaces of the body are covered with bacteria, and the highest concentration of microbial communities is found in the intestinal tract. Despite being an exposed mucosa, the eye is relatively free of bacteria. Routine cultures from conjunctival swabs were negative in more than 50% of cases [1,2,3]. This was thought to be due to the high concentration of antimicrobial peptides and proteins in the tear film [4,5,6,7,8,9]. Recent advances in metagenomics and bacterial sequencing have prompted the re-investigation of the ocular microbiome. Most of the recent literature has pointed to a very small bacterial load on the ocular surface and a non-existent “core” ocular microbiome [1,10,11,12,13,14,15]. Recent studies have also highlighted the importance of some eye commensal bacteria in the context of corneal infections in C57BL/6 mice [16,17,18,19].

In a previous study, we observed distinct differences in the oral and stool microbiome of Sjögren syndrome patients compared to healthy controls, characterized by a decreased microbial richness in the mouth and intestine and significant differences in some genera in both mucosal sites [12]. We also observed that low bacterial diversity in the gut inversely correlated with total disease severity. Furthermore, we showed that antibiotic treatment worsened dry eye disease parameters in mice subjected to desiccating stress [12].

There is a great body of evidence indicating that the gut microbiome my influence autoimmunity at distant sites, such as the lung and brain [20,21,22,23,24]. Reduced diversity of the stool microbiome in antibiotic-treated mice has been found to increase the severity of response to inflammatory stimuli such as osmotic stress of colonic mucosa of mice with dextran sulfate sodium (DSS) [25]. Studies have shown that commensal bacteria in the gut have immunomodulatory functions affecting natural killer cells, natural killer T cells, dendritic cells and effector and regulatory T cells (Tregs) [26,27].

The purpose of this study was to investigate whether commensal bacteria participate in maintenance of ocular surface homeostasis, therefore establishing a gut-ocular surface-lacrimal gland axis. We demonstrate that germ-free C57BL/6J mice have a spontaneous Sjögren-like lacrimal keratoconjunctivitis, with autoreactive T cells that can transmit the disease phenotype. Fecal microbiota transplant from conventionally housed mice into germ-free mice improved corneal barrier and conjunctival goblet cell density and decreased the frequency of Th1 cells, indicating that commensal bacteria have an essential function in maintaining immune homeostasis at the ocular surface. Possible mechanisms by which intestinal bacteria ocular surface impact immune tone or induce pathology will be discussed.

## 2. Results

### 2.1. Spontaneous Sjögren-Like Lacrimal Keratoconjunctivitis

**Figure 1:**
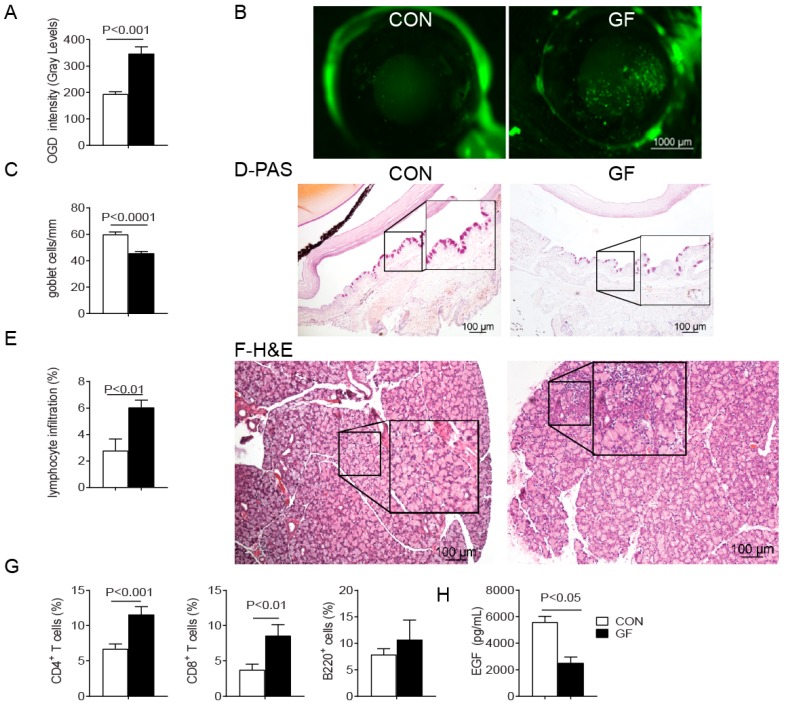
Sjögren-like Lacrimal Keratoconjunctivitis on germ-free mice. Conventionally housed mice with complex microbiota served as control (CON) and were compared to germ-free (GF) mice at 8 weeks of age. Both sexes were pooled. (**A**) Corneal Oregon-Green dextran fluorescence intensity score. Bar graphs show means ± SEM of two independent experiments with four to five animals per experiment (final *n* = 8–10 animals, mixed sex); (**B**) Representative images of corneas stained with Oregon Green dextran (OGD); (**C**) Number of Periodic acid–Schiff (PAS)^+^ conjunctival goblet cells counted in paraffin-embedded sections expressed as number per millimeter. Bar graph show means ± SEM of two independent experiments with three animals per group, yielding a final sample of six right eyes for each group); (**D**) Representative images of conjunctiva sections stained with PAS used to generate the bar graph in (**C**); (**E**) Total lacrimal gland infiltration measured in haematoxylin and eosin (H&E) stained sections as shown in (**F**) (*n* = 6 right lacrimal gland); (**F**) Representative images of H&E-stained sections of lacrimal gland. Black rectangular insets are a high magnification of dotted square; (**G**) Flow cytometric analysis of CD4^+^ and CD8^+^ T cells and B220^+^ B cells in lacrimal gland. Right and left extraorbital lacrimal glands from one mouse per group were excised and pooled into a single tube, yielding a final sample of 12 individual lacrimal gland samples divided into two independent experiments with six samples per experiment. Bar graphs show means ± SEM; (**H**) Tear epidermal growth factor (EGF) concentrations were measured by enzyme-linked immunosorbent assay. Tear washings from both right and left eyes from one mouse per group were collected and pooled into a single tube, yielding a final sample of 12 individual samples per group and divided into three independent experiments with four samples per experiment) Mann-Whitney U test was used for germ-free vs. conventional mice comparisons.

Sjögren syndrome is an autoimmune disease with a recognized female sex predilection [28,29]. Hallmarks of Sjögren syndrome related lacrimal keratoconjunctivitis include corneal barrier disruption, conjunctival goblet cell (GC) loss, lymphocytic infiltration and epithelial apoptosis in the lacrimal gland [30,31]. Conventionally housed C57BL/6J with complex microbiota and germ-free C57BL/6J mice of both sexes were examined for a dry eye phenotype. To accomplish this, we investigated the uptake of a fluorescent dye as a measurement of corneal permeability and examined Periodic acid–Schiff (PAS)-stained histologic sections from the conjunctival to measure conjunctival goblet cell density. Increased corneal permeability to the fluorescent tracer dye indicative of corneal barrier disruption was found in germ-free mice compared with conventional mice (Figure 1A–D). A significant decrease in the number of mucin-filled conjunctival GCs was also observed in the germ-free mice (Figure 1C,D).

Histological analysis of the lacrimal glands showed greater lymphocytic infiltration in germ-free lacrimal gland compared to conventional mice (Figure 1E,F); however infiltration of the submandibular salivary glands was not observed in either group. Flow cytometry analyses indicated that immune cell infiltrates in the germ-free lacrimal gland consisted of greater frequencies of CD4^+^, and CD8^+^ T cells, but not B lymphocytes (B220^+^) (Figure 1G). Epidermal growth factor (EGF) is secreted by lacrimal glands into tears, is measurable in the tears of mice, and significantly lower tear EGF concentrations have been found in murine models of Sjögren syndrome [32,33]. Lacrimal gland inflammation in germ-free mice was accompanied by a significant decrease in tear EGF levels, compared to conventional mice (Figure 1H). These results indicate that 8-week old germ-free mice spontaneously developed a Sjögren-like lacrimal keratoconjunctivitis.

### 2.2. Sex Differences in Ocular Surface Manifestations

Data were segregated by sex to assess differential ocular surface and lacrimal gland immunophenotypes (Table 1). Greater corneal barrier disruption and greater total lacrimal gland infiltration were noted in females, while similar levels of goblet cell loss, frequency of CD4^+^ and CD8^+^ T cell infiltration and tear EGF concentration were observed in both sexes.

In agreement with greater infiltration observed in female germ-free lacrimal glands greater levels of *MHC II*, *IFN-γ*, *IL-12* and *Caspase 3* mRNA transcripts were noted in female germ-free lacrimal gland lysates evaluated by real-time PCR, compared to male germ-free and male and conventionally housed female mice (Table 1). These markers were chosen because they have been shown to participate in development of SS dacryoadenitis [31,34,35]. Caspase 3 activity levels were also measured in lacrimal gland lysates and mirrored the PCR results (Table 1).

These tests indicate that a germ-free environment promotes similar conjunctival disease in both sexes, but more significant corneal disease and greater lacrimal gland pathology in female mice, recapitulating the sex predilection that is seen in Sjögren syndrome.

### 2.3. Changes in the Ocular Environment in Germ-Free Mice Predispose to Th-1 Cells

**Figure 2:**
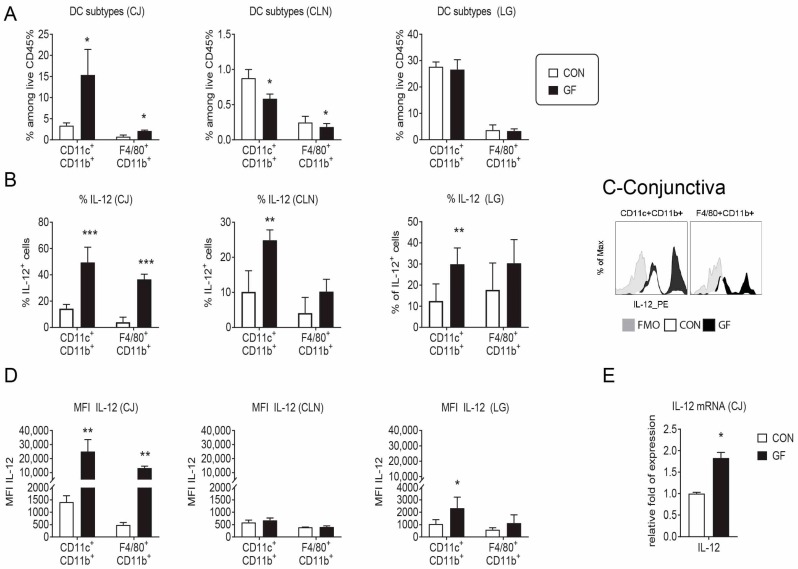
DC frequency and production of IL-12 is altered in germ-free mice. Single cell suspensions of the conjunctiva (CJ), cervical lymph nodes (CLN) and lacrimal glands (LG) were prepared and stained for antigen-presenting cell and macrophage markers followed by intracellular staining for IL-12. Bar graphs are shown as means ± SEM of a representative experiment with 4–5 samples per group. The experiment was repeated once with similar results. (**A**) Accumulative data showing frequency of CD11b^+^CD11c^+^ and F4/80^+^CD11b^+^ cells among alive CD45^+^ gated cells; (**B**) Accumulative data showing frequency of IL-12^+^ among viable CD45^+^ gated cells; (**C**) Representative overlaid histogram of IL-12 staining in conjunctival CD11b^+^CD11c^+^ and F4/80^+^CD11b^+^ cells; (**D**) Accumulative data showing median fluorescence intensity (MIF) of IL-12 in positive cells; (**E**) Relative fold expression changes of IL-12 in full-thickness conjunctival biopsies. Bar graphs show means ± SD of five samples per group, biological replicates from two independent experiments were averaged. FMO = fluorescence minus one; GF = germ-free; CON = conventional mice; * *p* < 0.05; ** *p* < 0.01; *** *p* < 0.001. Mann–Whitney U test was used for GF vs. CON comparison test.

Since disease parameters were more severe in the female sex, we opted to continue the experiments using only females. Goblet cells secrete mucins but also immunoregulatory factors such retinoic acid and TGF-β that can modulate dendritic cells [36,37]. Moreover, mice devoid of goblet cells have increased conjunctival inflammation and loss of conjunctival tolerance [38,39]. The Th1 cytokine IFN-γ causes goblet cell apoptosis, an unfolded protein response and reduced secretory response to cholinergic agonists [40,41]. Since germ-free mice displayed a 40% reduction in conjunctival goblet cells compared to age-matched conventional mice, we then investigated the frequency of antigen-presenting cells and their ability to produce the Th1-inducing cytokine IL-12 in the conjunctiva, cervical lymph nodes (CLN) and lacrimal gland using flow cytometry analysis. A significant increase in the frequency of CD11b^+^CD11c^+^ and F4/80^+^CD11b^+^ cells was present in germ-free conjunctiva, while a decrease in these cells was seen in draining CLN (Figure 2A). This was accompanied by an increased number of IL-12^+^CD11b^+^CD11c^+^ cells in conjunctiva, CLN and lacrimal gland (Figure 2B,C). On a per a cell basis, increased IL-12 median-fluorescence intensity (MIF) was present in conjunctiva CD11b^+^CD11c^+^ and F4/80^+^CD11b^+^ cells and lacrimal gland CD11b^+^CD11c^+^ cells. (Figure 2D). A 2-fold increase in number of IL-12a mRNA transcripts was found in full-thickness conjunctival biopsies from female germ-free compared to conventional mice (Figure 2E).

These results indicate that germ-free environment increases frequency and production of IL-12 by antigen presenting cell in the lacrimal gland functional unit.

### 2.4. CD4^+^ T Cells from Germ-Free Mice Induce Sjögren-Like Lacrimal Keratoconjunctivitis

**Figure 3:**
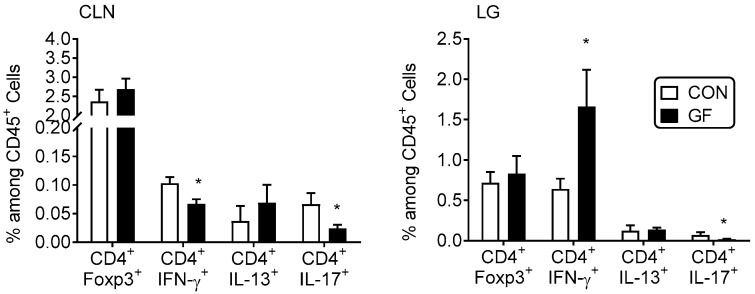
Increased percentage of Th1^+^ cells in lacrimal gland of germ-free mice. Percentage of CD4^+^Foxp3^+^, Th1 (CD4^+^IFN-γ^+^), Th2 (CD4^+^IL-13^+^), Th17 (CD4^+^IL-17^+^) cells in freshly isolated cervical lymph nodes (CLN) and lacrimal glands (LG) by flow cytometry. Each experiment group consisted of three-to-four samples; showing mean ± SEM of two independent experiments (final *n* = seven to eight per group). CLN of the same animal were pooled; right and left extraorbital LGs of the same animal were pooled. * *p* < 0.05 conventional vs. germ-free comparison using Mann-Whitney U test.

Next, we surveyed the frequency of CD4^+^Foxp3^+^ regulatory T cells and Th-1, -17 and -2 cells by flow cytometry in lacrimal gland and CLN suspensions. Although a significant decrease in Foxp3^+^Tregs has been previously noted in colonic lamina propria of germ-free and antibiotic-treated B6 mice [42], there was no difference in the frequency of CD4^+^Foxp3^+^ T cells between the conventional and germ-free mice in both lacrimal gland and CLN (Figure 3). A decreased frequency of CD4^+^IFN-γ^+^ and CD4^+^IL-17^+^ cells was observed in the CLN, while a significant increase in Th1^+^ cells was seen within the lacrimal gland (Figure 3). These results suggested that effector T cells may accumulate at the Sjögren syndrome target organ (i.e., lacrimal gland).

**Figure 4:**
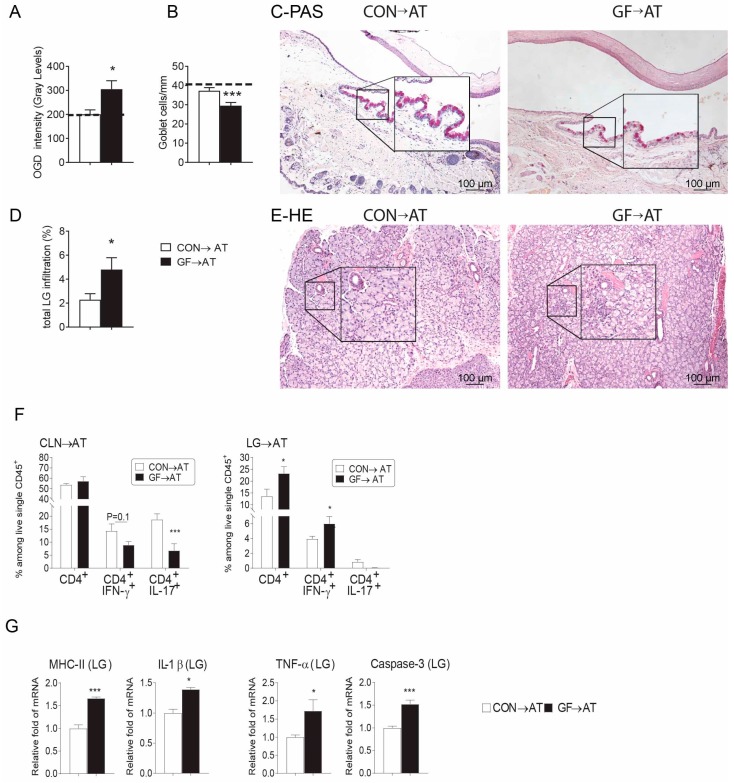
CD4^+^T Cells from germ-free mice transfer Sjögren-like lacrimal Keratoconjunctivitis to immunodeficient RAG1KO mice. CD4^+^T cells were isolated from spleens and cervical lymph nodes (CLN) from conventionally housed (CON) and germ-free (GF) mice and adoptively transferred (→AT) into RAG1KO mice. Disease severity parameters were evaluated five weeks later. Bar graphs show means ± SEM of a representative experiment with 18–20 samples per group. (**A**) Corneal barrier function measured by Oregon-Green dextran fluorescence intensity score. Bar graphs show means ± SEM of two independent experiments with four animals per experiment (final *n* = eight animals, female sex). Dotted line demonstrates OGD fluorescence intensity score in naïve RAG1KO mice; (**B**) Number of PAS^+^ conjunctival goblet cells counted in paraffin-embedded sections expressed as number per millimeter. Bar graphs show means ± SEM of two independent experiments with three animals per group, yielding a final sample of six right eyes for each group. Dotted line demonstrates goblet cell density in naïve RAG1KO mice; (**C**) Representative images of conjunctiva sections stained with PAS (purple cells) used to generate the bar graph in (**B**). Black rectangular insets are a high magnification of the small demarcated area; (**D**) Total lacrimal gland infiltration measured in H&E stained sections as shown in (**F**) (*n* = six right lacrimal glands); (**E**) Representative images of haematoxylin and eosin (H&E)-stained sections. Black rectangular insets are a high magnification of small demarcated area; (**F**) Percentage of total CD4^+^ T, Th1 (CD4^+^IFN-γ^+^), Th2 (CD4^+^IL-13^+^), Th17 (CD4^+^IL-17^+^) cells in freshly isolated cervical lymph nodes (CLN) and lacrimal glands (LG) by flow cytometry five weeks post-adoptive transfer. Each experiment group consisted of three-to-four samples; showing mean ± SEM of two independent experiments. CLN of the same animal were pooled; right and left extraorbital LGs of the same animal were pooled. Bar graphs show means ± SEM of two independent experiments with four animals per experiment (final *n* = eight animals, female sex); (**G**) Relative fold expression changes of *MHC II, IL*-*1β*, *TNF*-*α* and *Caspase 3* in LG. Bar graphs show means ± SD of six samples per group, biological replicates from two independent experiments were averaged. * *p* < 0.05; *** *p* < 0.001Mann-Whitney U GF vs. CON comparison test.

We have previously demonstrated that adoptive transfer (AT) of either desiccated-activated or aged CD4^+^T cells induces dry eye disease in naïve T cell deficient hosts [30,43,44,45]. Using this strategy, we performed AT experiments by transferring isolated CD4^+^ T cells from CLN and spleens from both conventional and germ-free mice i.p. into female RAG1KO mice. Induction of dry eye disease parameters was investigated in recipients five weeks post-injection. Female germ-free CD4^+^ T cell recipients had greater corneal barrier disruption, goblet cell loss, and lacrimal gland total lymphocytic infiltration (Figure 4A–E), recapitulating the disease phenotype in donor female germ-free mice (Table 1). Similar to the donor mice, adoptive transfer recipients of germ-free cells had a lower frequency of Th1^+^ and Th17^+^ cells in CLN while a significant increase in Th1^+^ cells was present in the lacrimal gland (Figure 4F). Lacrimal gland lysates of germ-free recipients showed increased mRNA levels of *MHC II*, *TNF*-*α*, *IL*-*1β*, and *Caspase 3* compared to conventional mice, indicating the cytokine milieu inside the lacrimal gland became pro-inflammatory after adoptive transfer of germ-free CD4^+^ T cells (Figure 4G).

### 2.5. Fecal Microbiota Transplant Reversed Lacrimal Keratoconjunctivitis in Germ-Free Mice

**Figure 5:**
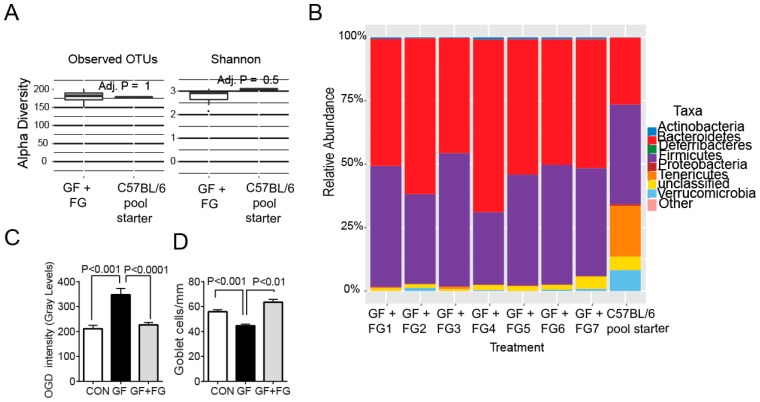

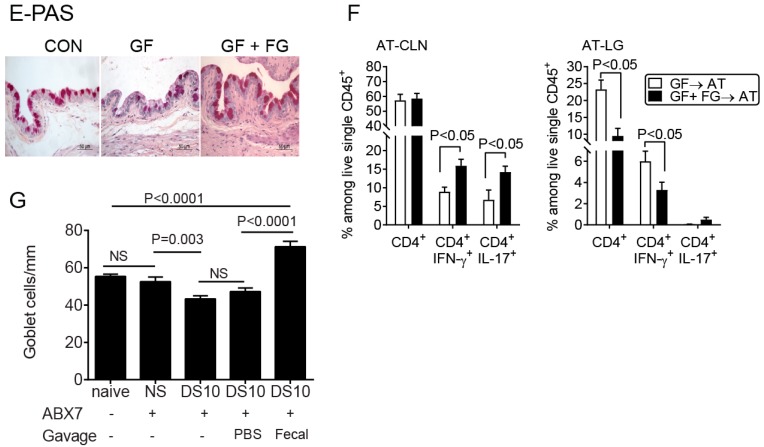
Reconstitution of germ-free mice with commensal bacteria reverses the dry eye phenotype. (**A**–**C**) Four-week-old female germ-free mice were colonized with a fecal slurry from normal mice (a pool of three mice) by intragastric gavage and sacrificed at eight weeks of age (germ-free + Fecal gavage, GF + FG). (**A**) Comparison of α diversity between starting inoculum (C57BL/6J) and germ-free conventionalized mice (*n* = seven) using measures of observed OTUs and Shannon’s diversity index; (**B**) Relative abundance of major bacterial phyla in the starting inoculum (C57BL/6J pool) used to conventionalize mice and in the gut microbiota from conventionalized germ-free mice that received fecal gavage (*n* = seven) at eight weeks of age; (**C**) Corneal Oregon-Green dextran fluorescence intensity score. Bar graphs show means ± SEM of two independent experiments with four animals per experiment (final *n* = eight animals, female sex). Mann-Whitney U comparison test; (**D**) Number of PAS^+^ conjunctival goblet cells counted in paraffin-embedded sections expressed as number per millimeter. Bar graphs show means ± SEM of two independent experiments with three-four animals per group, yielding a final sample of seven right eyes for each group). Mann-Whitney U comparison test; (**E**) Representative images of conjunctiva sections stained with PAS used to generate the bar graph in (**D**); (**F**) Flow cytometry analysis showing the percentage of total CD4^+^, Th1 (CD4^+^IFN-γ^+^), Th17 (CD4^+^IL-17^+^) cells in freshly isolated cervical lymph nodes (CLN) and lacrimal glands (LG) in adoptive transfer (→AT) recipients of either germ-free or germ-free + FG CD4^+^T cells. Each experiment group consisted of three-to-four samples; showing means ± SEM of two independent experiments, yielding a final sample of seven to eight per group. Mann-Whitney U comparison test; (**G**) Fecal transplant during desiccating stress (DS) rescues goblet cells. Female conventional C57BL/6 mice were left untreated (naïve mice) or received a cocktail of antibiotics (ABX) for seven days. On the morning of the 8th day, mice were switched to normal water and subjected to desiccating stress (DS) for ten days (DS10) and randomized to receive either PBS or oral gavage of fecal material or were left non-stressed. Mice under DS were sacrificed after ten days, and the number of PAS^+^ cells in the conjunctiva was determined. *n* = five animals/group. Kruskal-Wallis test followed by Tukey’s post hoc test. NS = non-significant.

Our published results have shown that both in SS patients and in the murine desiccating model low intestinal microbial diversity correlates with the more severe disease phenotype [12]. This is in agreement with reports that oral antibiotic treatment exacerbates mucosal inflammation and that intestinal recolonization with commensal bacteria reverses inflammatory changes [25]. To determine if intestinal recolonization could reverse the dry eye phenotype observed in germ-free mice we performed two experiments. In the first set of experiments, four-week-old female germ-free mice were colonized with fecal material from normal mice, and disease parameters were evaluated at eight weeks of age. Microbial ecology analysis of bacterial communities after fecal gavage demonstrated germ-free animals were colonized with complex microbiota (Figure 5A,B). Measures of alpha diversity indicated that there was no significant difference between the starting inoculum (*n* = 182) and the amount of OTUs present (*n* = 178) in conventionalized mice suggesting a high efficiency of microbiota transfer (Figure 5A). The Simpson diversity index was also similar between the inoculum and conventionalized mice. 16S rRNA sequencing demonstrated that the relative abundance of the major bacterial phyla from the fecal samples of conventionalized mice closely reflected the microbial composition in the starting inoculum as Firmicutes and Bacteroidetes accounted for a majority of the sequences (Figure 5B). Germ-free mice that received the fecal material gavage had minimal corneal barrier disruption, indicating that corneal epithelial barrier was normalized (Figure 5C). Similarly, goblet cell density was significantly improved (Figure 5C,D). Adoptive transfer recipients of CD4^+^ T cells isolated from germ-free mice that received fecal transplant had lower total CD4^+^ T cell infiltration and lower frequency of Th1^+^ cells in the lacrimal gland, demonstrating that colonization with commensals decreased generation of autoreactive CD4^+^ T cells (Figure 5E).

In another set of animals, we performed fecal microbiota transplantation in mice subjected to an experimental desiccating stress (DS) dry eye model that had been subjected to a cocktail of oral antibiotics seven days before initiation of DS. Mice received oral gavage of either PBS or fecal material daily for five days starting at day 1 of DS, and they were euthanized at day +10, a time point where significant goblet cell loss is typically observed in this model [44,46,47]. Mice that received fecal transplant during DS had a 50% increase in goblet cells compared to mice that received PBS gavage, demonstrating that the protective role of microbiota on conjunctival goblet cell (Figure 5F).

These results demonstrate that corneal barrier disruption and low goblet cell density were related to lack of bacterial colonization of the gut, indicating that commensal bacteria participate in the maintenance of ocular surface homeostasis.

## 3. Discussion

Sjögren syndrome is a chronic autoimmune disease characterized predominantly by lymphocyte infiltration of the lacrimal and salivary glands. This study is the first to demonstrate germ-free mice spontaneously develop Sjögren-like lacrimal keratoconjunctivitis with corneal barrier disruption, reduced conjunctival goblet cell density, lower tear EGF concentration and increased lymphocytic infiltration inflammatory cytokine expression in the lacrimal gland. Interestingly, female mice had an overall worse dry eye phenotype than male germ-free mice, suggesting that female sex in germ-free environment recapitulates the sex predilection seen in human SS patients [29].

Germ-free mice live longer, weigh less, and show decreased basal metabolic rate than mice with conventional microbiota [48,49]. In agreement with our findings of significant goblet cell loss, it has been reported that germ-free mice have fewer and smaller goblet cells in the cecum than conventional mice and that recolonization improves their number [50]. Our study adds the conjunctiva to the list of mucosal sites with reduced goblet cell density when raised in germ-free conditions. These findings suggest microbiota play a critical role in systemic goblet cell development. However, exact mechanisms by which microbiota regulate the goblet cell development are still largely unknown.

Our results showed that lacrimal gland lysates of germ-free mice had increased *MHC II* and *IFN-γ* mRNA transcripts. It is accepted that autoantigens presented by MHC class II antigens to CD4^+^ T cells play a crucial role in the early stage pathogenesis of SS [51]. In mice with TGF-β mutation, enhanced expression of MHC II was detected, as well as SS-like lymphoproliferation in multiple exocrine glands. In TGF-β1/MHC II double knockout mice, this lymphocytic infiltration was not observed, indicating MHC II plays a significant role in the pathogenesis of SS-like exocrinopathy [52]. IFN-γ is a critical cytokine that is involved in acini loss in salivary and lacrimal exocrine glands [31,53,54,55,56]. IFN-γ also causes goblet cell loss and induces an unfolded protein response and suppresses cholinergic agonist-induced mucin secretion [40,41,46,57,58,59]. Other cytokines involved in SS include BAFF, IL-6, IL-23, TNF-α, and IL-1β [60,61].

Since a greater disease phenotype was observed in germ-free mice, we asked if a decrease in regulatory T cells or increased pathogenicity on a per cell basis could be responsible for the dry eye phenotype. A significant decrease in Foxp3^+^ cells has been noted in colonic lamina propria of germ-free mice; antibiotic-treated B6 mice and conventionalization of germ-free with feces from normal mice restored the typical frequency of Foxp3^+^ Tregs, indicating that signals from microbiota modulate Treg numbers in the gut [42,62]. However, our study found an equal frequency of CD4^+^Foxp3^+^ cells in both cervical lymph nodes and lacrimal glands of both germ-free and conventional mice, indicating that in contrast to the gut, other mechanisms might be at play in the ocular surface. This is important because it shows that microbial communities may promote ocular health through a different mechanism than the gut and it warrants further investigation.

Our adoptive transfer experiments demonstrated that recipients of CD4^+^ T cells from germ-free donors recapitulated the lacrimal keratoconjunctivitis phenotype that was observed in the germ-free donors. This was not observed in recipients of CD4^+^ T cells from conventionally housed donors. Interestingly, recipients had a Th-1 predominant phenotype, with increased IFN-γ production. The pathogenic role of IFN-γ in inducing apoptosis of glandular acini in Sjögren syndrome has been well documented in the literature [31,53,54,63,64,65,66,67,68,69,70,71]. These findings highlight that a lack of commensal bacteria or products secreted by them promotes the development of pathogenic Th1 cells, but decreased frequency of Tregs is not part of this mechanism.

Fecal material transplant has recently as a therapeutic option to treat inflammatory bowel disease developing from *Clostridium difficile* infestations [72,73,74], but it has become evident that the microbiome may modulate inflammation in tissues distant from the intestinal tract, such as the brain and the lung [20,21,22,23,24]. Our findings indicate that fecal material transplant restored goblet cell density and corneal barrier function in germ-free mice and mice subjected to desiccating stress indicating that a “gut-ocular surface axis” also exists. Further studies are necessary to delineate the exact bacterial species or bacterial communities that are responsible for maintenance of ocular surface homeostasis. Manipulation of intestinal commensal bacteria thus has the potential to become a novel therapeutic approach to treat Sjögren Syndrome.

## 4. Methods

### 4.1. Animals

The research protocol was approved by the Baylor College of Medicine Institutional Animal Care and Use Committee (IACUC protocol number AN-6491, first approved on 27 February 2014), and it conformed to the standards of the ARVO Statement for the Use of Animals in Ophthalmic and Vision Research. Germ-free C57BL/6J (B6) mice were bred and maintained in flexible-film isolators (Class Biologically Clean, Madison, WI, USA) at Baylor College of Medicine and were compared to specific pathogen-free C57BL/6J mice with a complex microbiota. Germ-free mice receive autoclaved LabDiet 5V0F. Regular mice receive irradiated LabDiet 5V5R LabDiet products are manufactured by PMI Nutrition International, St. Louis, MO, USA. Both groups received water ad libitum, which was sterilized for the animals housed in germ-free conditions. Conventionally housed RAG1KO (Recombination activating gene 1; B6.129S7-*Rag1*^tm1Mom/^J) breeding pairs were purchased from The Jackson Laboratory (Bar Harbor, ME, USA) for establishing breeding colonies and raised under specific pathogen-free conditions at Baylor College of Medicine. For these experiments, 30 male (either conventional of germ-free) and 105 female (either conventional or germ-free) B6 mice were used; while 40 female RAG1KO mice were used for adoptive transfer experiments. In the present study, we chose to investigate 8-week old mice, as C57BL/6J mice older than 9 months develop a spontaneous dry eye phenotype [30].

### 4.2. Corneal Permeability

Corneal epithelial permeability to Oregon green dextran (OGD; 70,000 MW; Invitrogen, Eugene, OR, USA) was assessed in eight-week-old germ-free mice and conventional mice of both sexes as previously published [75]. Briefly, 0.5 µL of 50 mg/mL OGD was instilled onto the ocular surface 1 min before euthanasia. The severity of corneal OGD staining was graded in digital images in the 2-mm central zone of each cornea by two masked observers, using NIS Elements Software (Nikon, Inc., New York, NY, USA). The mean fluorescent intensity measured by the software inside this central zone was recorded in a database and results averaged within each group. Results are presented as means ± SEM of gray levels.

### 4.3. Histology and PAS Staining

Eyes with ocular adnexa and extraorbital lacrimal glands were surgically excised, fixed in 10% formalin, and embedded in paraffin, and 8-µm sections were cut using a microtome (Microm HM 340E, Thermoscientific Wilmington, DE, USA). Sections were stained with haematoxylin and eosin for evaluation of morphology. Goblet cells in sections were stained with periodic acid-Schiff (PAS) reagent and were examined, photographed and counted with a microscope equipped with a digital camera (Eclipse E400 with a DS-Fi1; Nikon, Inc., Melville, NY, USA) as previously described [75]. The number of positively stained goblet cells in the superior and inferior conjunctiva was counted, and the length of the basement membrane between the first and last goblet cell was measured. The data are presented as the average number of goblet cells per millimeter per mouse.

### 4.4. Measurement of Lacrimal Gland Infiltration

Total lymphocytic cell infiltration area was evaluated in digital images of H&E-stained sections of paraffin-embedded sections as previously described [31]. The percentage infiltration was calculated as the area of infiltration × 100/total area.

### 4.5. Flow Cytometry Analysis of Infiltrating Cells

Single-cell suspensions of cervical lymph nodes (CLN), and lacrimal glands from germ-free and conventional mice of both sexes were prepared as previously reported [31,76]. In brief, right and left extraorbital lacrimal glands were excised, rinsed, and subjected to collagenase digestion for 1 h at 37 °C under constant agitation. Collagenase was neutralized by adding complete RPMI with 10% fetal bovine serum; cells were filtered by using a 70 µm cell strainer, centrifuged, and resuspended. Cells were stained with anti-CD16/32 (BD Biosciences, San Jose, CA, USA), followed by cell-surface staining with anti-CD4-FITC (clone GK 1.5; BD Biosciences, San Diego, CA, USA), anti-CD8-PE (clone 53-6.7; BD Biosciences), anti-B220-APC (clone RA3-6B2; BD Biosciences). Propidium iodide exclusion was used to discriminate live cells. Cells were kept on ice until flow cytometry analysis was performed.

For intracellular cytokine staining, single cell suspensions were obtained and 1 × 10^6^ cells were incubated for five hours with 1 µL/mL Golgi Stop (BD Bioscience), 1 µL/mL Golgi Plug (BD Bioscience), PMA (1 µg/mL) (Sigma, St. Louis, MO, USA), ionomycin (1 µg/mL) (Sigma) in 1 mL in complete RPMI. Cells were stained with a blue fluorescent reactive dye (Life Technologies, Grand Island, NY, USA) for 30 min before incubation with Foxp3 Fixation/Permeabilization working solution (eBioscience, San Diego, CA, USA) for 18 h Cells were washed with 1X Permeabilization solution and incubated with anti-CD16/32, followed by staining with anti-CD4-FITC (clone GK1.5, BD Bioscience), IL-17-PE (clone eBio17B7, eBioscience, San Diego, CA, USA), and IL-13-eFluor^®^ 660 (clone eBio13A, eBioscience, San Diego, CA, USA), anti-IFN-γ-Pacific Blue (clone XMG1.2, Biolegend, San Diego, CA, USA), and anti-CD45-AlexaFluor 700 (clone 30F11, Biolegend, San Diego, CA, USA). The following gating strategy was used: dead cells were excluded by gating blue dye versus CD45^+^ cells, subsequently gated by forward scatter height versus forward scatter area (singlets 1), then gated on side scatter height versus side scatter area (singlets 2). Cells were then gated on CD4^+^ cells and CD4^+^ IFN-γ^+^ or CD4^+^IL-17A^+^ or CD4^+^IL-13^+^ cells were evaluated. All flow cytometry experiments used the BD LSRII Benchtop cytometer.

For IL-12 intracellular staining, single cell suspensions were obtained and 2 × 10^6^ cells were incubated for five hours with one µL Golgi Stop (BD Bioscience) and 1 µL Golgi Plug (BD Bioscience) in 1 mL in complete RPMI. Cells were stained with an infra-red fluorescent reactive dye (Life Technologies, Grand Island, NY, USA) for 30 min, before fixation. Cells were then stained with CD16/CD32 followed by incubation with anti-CD45-BV510 (clone 30F11, BD Biosciences), anti-CD11c-FITC (clone HL3, BD Biosciences), anti-IL-12-PE (p40/p70, clone C15.6, BD Biosciences), anti-F4/80-APC (clone BM8, Biolegend), and anti-CD11b-PE-Cy7 (clone M1/70, BD Biosciences). The gating strategy used in this study was as follows: dead cells were excluded by gating live dye versus CD45^+^ cells, subsequently gated by forward scatter height versus forward scatter area (singlets 1), then gated on side scatter height versus side scatter area (singlets 2). CD11b and CD11c were then plotted; IL-12 percentage and MFI was then calculated in each cell subset. A BD FACS CANTO II cytometer (Becton Dickinson, San Jose, CA, USA) was used.

Data were acquired with BD Diva software (version 2.1; BD Biosciences) and FlowJo software (version 10.1; Tree Star, Inc., Ashland, OR, USA). Biological replicates were averaged.

### 4.6. Tear Washings and EGF Enzyme-Linked Immunosorbent Assay

Tear-fluid washings were collected from each group from conventional and germ-free C57BL/6J mice of both sexes, and EGF concentration in tear samples was assayed with a commercial ELISA kit according to the manufacturer’s protocol (R&D Systems, Minneapolis, MN, USA) [31]. One sample consisted of tear washings from both eyes of one mouse pooled (2 μL) in PBS + 0.1% BSA (8 μL) and stored at −80 °C until the assay was performed. The microplate was read by using an ELISA-reader instrument (Tecan Infinite M200) equipped with Magellan V6.55 software. Biologic replicate samples were averaged. Results are presented as the mean ± standard deviation (pictograms per milliliter).

### 4.7. Caspase-3 Activation Fluorometric Assays

The activation of caspase-3 was measured in lacrimal gland lysates according to the manufacturer’s protocol (K105-200, BioVision, Inc., Mountain View, CA, USA) [31]. Protein concentration was measured using a micro BCA protein assay kit (Thermo Fisher Scientific, Waltham, MA, USA). Five to nine samples per group were used. Caspase-3 activities were measured (50 μg/sample) by following the cleavage of the fluorescent substrate analogs in a fluorescent plate reader (Tecan Infinite M200, Magellan V6.55 software; Tecan, Männedorf, Switzerland) with 400-nm excitation filter and 505-nm emission filter. The results were exported and averaged.

### 4.8. RNA Isolation and Real-Time PCR

Total RNA from lacrimal gland from germ-free and conventional mice was extracted by using RNeasy Plus Mini Kit (Qiagen RNA isolation kit; Qiagen, Inc., Valencia, CA, USA) according to the manufacturer’s instructions, quantified by a spectrophotometer (NanoDrop ND-1000; Thermo Scientific, Wilmington, DE, USA), and stored at −80 °C. Samples were treated with DNase (Qiagen, Inc.) to prevent genomic DNA contamination, according to the manufacturer’s instructions. First-strand complementary DNA was synthesized from 1 µg of total RNA using random hexamers and Ready-To-Go You-Prime First-Strand Beads; GE Healthcare, Arlington Heights, NJ, USA), as previously described [75]. Quantitative real-time PCR was performed with specific minor groove binder (MGB) probes (Taqman; Applied Biosystems, Foster City, CA, USA) and PCR master mix (Taqman Gene Expression Master Mix) in a commercial thermocycling system (StepOnePlus Real-Time PCR System; Applied Biosystems). Murine MGB probes were IFN-γ (*IFN-*γ, Mm00801778_m1), major histocompatibility complex class II (*MHC II*, Mm00482914_m1), tumor necrosis factor alpha (*TNF-α*, Mm00443258_m1), interleukin-1β (*IL-1β*, Mm00434228), caspase-3 (*casp3*, Mm00438045_m1), IL-12a (*IL-12a*, Mm00434165). The hypoxanthine phosphoribosyltransferase 1 (*HPRT-1*, Mm00446968_m1) gene was used as an endogenous reference for each reaction. The results of quantitative PCR were analyzed by the comparative *C*_t_ method in which the target of change = 2^−∆∆*C*t^ and were normalized by the *C*_t_ value of HPRT-1 and the mean *C*_t_ of relative mRNA level in the conventional female group at eight weeks of age was used as the calibrator.

### 4.9. Isolation of Murine CD4^+^ T Cells and Adoptive Transfer

Superficial cervical lymph nodes and spleens from donor mice were gently meshed between two frosted end slides, as previously described [43]. Ammonium chloride Tris was used for eliminating erythrocytes. Untouched CD4^+^ cells were isolated by negative selection using magnetic beads according to the manufacturer’s instructions (MACS system; Miltenyi Biotec, Auburn, CA, USA). Isolated cells were used in adoptive transfer experiments. The purity of CD4^+^ T cells was determined to be >90% by flow cytometry. CD4^+^ T cells were isolated from four to five mice per time point in four independent experiments using a total of 20 mice/group. Isolated CD4^+^ T cells (5 × 10^6^; approximately one donor-equivalent of cells) were intraperitoneally transferred to T cell-deficient RAG1KO female mice. Parameters of ocular surface disease were evaluated five weeks after adoptive transfer of CD4^+^ T cells.

### 4.10. Standard Desiccating Stress Model

Desiccating stress (DS) was induced in female C57BL/6J mice aged six-to-eight weeks by sterile subcutaneous injection of 0.5 mg/mL scopolamine hydrobromide (Sigma-Aldrich, St. Louis, MO, USA) QID into alternating flanks and exposure to a drafty low humidity (<30% relative humidity) environment for 10 days (DS5 and DS10 respectively) as previously described [75]. Non-dry eye (non-stressed) mice, served as controls and were housed in a regular room with normal humidity (>30–50%).

### 4.11. Antibiotic Treatment and Desiccating Stress

Six-to-eight week old female C57BL/6J mice (Jackson Labs, Bar Harbor, ME, USA) were treated with a cocktail of broad-spectrum antibiotics (0.5 mg/mL Ampicillin (Dava Pharmaceuticals, Fort lee, NJ, USA), 0.5 mg/mL Gentamicin (Life tech; Grand Islands, NJ, USA), 0.5 mg/mL Metronidazole (Hospira; Lake Forest, IL, USA), 0.5 mg/mL Neomycin (Sparhawk lab; Lenexa, KS, USA), 0.25 mg/mL Vancomycin (Hospira; Lake Forest, IL, USA)) dissolved in drinking water with 5 mg/mL artificial sweetener (Splenda™, McNeil Nutritionals; Fort Washington, PA, USA) as previously described [77]. For this experiment, antibiotic treatment started seven days before DS and mice drank regular water during DS because continuation of antibiotic during DS would affect fecal reconstitution and survival of newly transplanted bacteria. Mice were euthanized at day ten post-DS.

### 4.12. Fecal Microbiota Transplant

Cecal contents or stool pellets were collected from conventionally-raised C57BL/6J mice following euthanasia with 5% isoflurane until cessation of breathing. Before use, samples were resuspended in 25% *w*/*v* phosphate buffered saline, vortexed for 5 min at 2500 revolutions per minute (rpm) on a plate shaker followed by a centrifugation step for 5 min at 200× *g*. The supernatant portion was used to gavage 4-week old germ-free or desiccating-stressed animals (100 μL/mouse). All the remaining material was saved and stored in 20% glycerol at −80 °C for sequencing and 16S rRNA analysis. Animals were sacrificed at 4-weeks post gavage (germ-free mice) or at the 10th day of desiccating stress.

### 4.13. DNA Extraction from Mouse Fecal Samples, 16S rRNA Gene Amplication, and Sequencing

For 16S rRNA gene sequencing, DNA was extracted by bead beating followed by the use of the QIAGEN DNEasy Tissue Kit as previously described [78]. PCR Amplification of the V4 region of the 16S rRNA gene was performed with Phusion High Fidelity DNA Polymerase (New England Biolabs, Ipswich, MA, USA) using previously described primers and protocol [79,80]. PCR reactions were performed in duplicate. Replicates were pooled and cleaned using the Agencourt AMPure XP kit (Beckman Coulter, Brea, CA, USA). DNA sample concentrations were measured using Quant-iT (Life Technologies, Carlsbad, CA, USA) and pooled at equimolar ratios. Sequencing was performed on an Ilumina MiSeq platform.

### 4.14. Microbial Community Analysis

The MiSeq pipeline in Mothur was used to process sequence data [81]. The MiSeq pipeline for Mothur (essentially as described [82] and the MiSEQ SOP version 28 March 2013 (http://www.mothur.org/wiki/MiSeq_SOP) was used to process sequence data. Following alignment of forward and reverse reads, sequences were quality-trimmed and aligned to the Silva 16S rRNA gene reference database formatted for mothur. Sequences were then trimmed to overlap the same region of the 16S rRNA gene, pre-clustered to clusters with ≥99% identity, and potentially chimeric sequences were identified and removed using the mothur-impelementation of uchime. Sequences were classified according to the mothur-formatted ribosomal database project version 9 (August 2013) using the Bayesian classifier in Mothur, and those sequences classified as Eukarya, Archaea, Chloroplast, Mitochondria or unknown were removed. The sequence data was then filtered to remove any sequences present only once in the dataset. After building a distance matrix from the remaining sequences with the default parameters in mothur, sequences were clustered into operational taxonomic units (OTUs) with ≥97% using the average-neighbor algorithm in Mothur. Taxonomic assignments for each OTU are the majority consensus taxonomic assignment for each sequence within the OTU. Before analysis with the phyloseq package of R, additional filtering of the OTU table was done to remove rare OTUs. Namely, those OTUs present in less than three samples and that contained less than 25 sequences were removed. These filtering steps reduced the number of OTUs from 6306 to 923 but only decreased the number of sequences per sample by a mean of 0.7 ± 0.4% (range, 0.1–2.3%). Analysis and visualization of microbiome communities were conducted in R utilizing the phyloseq package to import sample data and calculate α- and β-diversity metrics [83].

### 4.15. Statistical Analysis

After completion of all experiments, data were averaged and graphs were generated. Sample size calculation was performed with StatMate Software version 2.0 (GraphPad Inc., San Diego, CA, USA), based on preliminary data. Data were expressed as a mean ± standard error of the mean (SEM). The data consisting of two groups were analyzed by two-tailed Student’s *t*-test or Mann-Whitney U test. Two-way ANOVA with Bonferroni post hoc testing adjusted for repeated measures was used for statistical comparison of multiple groups. *p* ≤ 0.05 was considered statistically significant. These tests were performed using GraphPad Prism 7.0 software (GraphPad Software, Inc., San Diego, CA, USA).

## Figures and Tables

**Table 1:** Summary of Findings in Ocular Surface and Lacrimal Gland within mice raised in conventional vivarium (CONV) or in germ-free (GF) conditions.

Parameters	Sex		CON		GF		*p* Value	
			F	M	F	M	CON vs. GF	F vs. M (GF)
Ocular surface	OGD intensity (gray levels)		241 ± 75.6	228.2 ± 71.5	344 ± 118	224 ± 147	↑, *p* < 0.001 (F)	F > M (*p* < 0.01)
	Goblet cell (cells/mm)		56.5 ± 8.8	53.2 ± 09.5	46.2 ± 7.4	43.0 ± 7.0	↓, *p* < 0.001 (F,M)	
	EGF (pg/mL)		4282 ± 1288	6667 ± 1787	2250 ± 750	2257 ± 167	↓, *p* < 0.05 (F,M)	
LG	Total LG infiltration (%) *		3.5 ± 3.0	1.38 ± 0.4	6.36 ± 1.7	4.07 ± 2.2	↑, *p* < 0.05 (F,M)	F > M (*p* < 0.05)
	CD4^+^ T cells (%) (flow cytometry)		6.0 ± 2.8	7.5 ± 3.7	10.9 ± 4.2	11.8 ± 4.8	↑, *p* < 0.05 (F,M)	
	CD8^+^ T cells (%) (flow cytometry)		4.63 ± 1.2	3.1 ± 0.9	10.1 ± 0.6	7.8 ± 2.3	↑, *p* < 0.001 (F,M)	
	PCR (fold)	IFN-γ	1.00 ± 0.25	0.96 ± 0.11	2.84 ± 0.57	1.31 ± 0.23	↑, *p* < 0.001 (F)	F > M (*p* < 0.05)
		MHC II	1.00 ± 0.09	0.92 ± 0.16	1.57 ± 0.10	0.89 ± 0.30	↑, *p* < 0.001 (F)	F > M (*p* < 0.05)
		Caspase 3	1.00 ± 0.04	0.57 ± 0.07	1.51 ± 0.10	1.00 ± 0.08	↑, *p* < 0.001 (F)	F > M (*p* < 0.001)
							↑, *p* < 0.01 (M)	
		IL-1β	1.00 ± 0.21	0.93 ± 0.03	0.76 ± 0.18	0.61 ± 0.14	↓, *p* < 0.05 (F,M)	
		IL-12	1.00 ± 0.08	1.00 ± 0.07	2.51 ± 0.7	2.28 ± 0.04	↑, *p* < 0.05 (F,M)	F > M (*p* < 0.01)
		TNF-α	1.00 ± 0.04	0.29 ± 0.21	0.80 ± 0.10	0.55 ± 0.12	Ø	Ø
	Caspase 3 activity (OD)		2355 ± 871	2754 ± 687	7499 ± 693	4110 ± 1201	↑, *p* < 0.001 (F)	F > M (*p* < 0.001)
							**↑** **, *p* < 0.01 (M)**	

OGD = Oregon-Green-Dextran; EGF = epidermal growth factor; F = female; M = male; LG = lacrimal gland; Ø = no change; * measured in histologic sections. ↑ = increase; ↓ = decrease.
